# Repetitive behaviors and life-quality in adults with autism spectrum disorder

**DOI:** 10.1192/j.eurpsy.2023.1009

**Published:** 2023-07-19

**Authors:** V. Johansson, S. Sandin

**Affiliations:** 1 Department of Clinical Sciences, Psychiatry Unit, Umeå University, Umeå; 2 Department of Medical Epidemiology and Biostatistics, Karolinska Institutet, Stockholm, Sweden; 3 Seaver Autism Center for research and treatment; 4 Department of Psychiatry, Icahn School of Medicine at Mount Sinai, New York, United States

## Abstract

**Introduction:**

Autism spectrum disorder (ASD) is characterized by deficits in social communication skills and repetitive behavior patterns. Lower quality of life has been reported by adults with high functioning ASD (Barneveld et al. CP, 2015; 55, 302–310, Dijkhuis et al. Autism, 2017; *21*, 896–906, Mason et al. AR, 2018; 11, 1138–1147). Less is however known about which autistic core symptoms are associated with lower life quality. One previous study found that quality of life was lower in adults with ASD who reported more repetitive symptoms during childhood (Moss et al. JADD, 2017; 47, 1830–1837). We therefore aimed to explore the relationship between repetitive symptoms and quality of life in adult ASD.

**Objectives:**

We present preliminary data on the relationship between self-reported repetitive behaviors and quality of life in a cohort of adults with a diagnosis of ASD. Our hypothesis is that higher levels of repetitive symptoms are associated with lower quality of life.

**Methods:**

We recruited 87 individuals, with a diagnosis of ASD, from a psychiatric out-patient clinic in Stockholm County. Mean age was 39.2 years and 52 females, 34 males, and one non-binary participated. The patients were also included in the *Population-Based Autism Genetics and Environmental study* (Pages) in Sweden. The participants answered a survey with questions regarding socio-demographics. They also completed self-assessment forms on repetitive behavior; *The Adult Repetitive Behaviors Questionnaire-2* (RBQ-2A, Barrett et al. JADD, 2015; 45, 3680–3692), and quality of life; *Diener Satisfaction with Life scale (DSWLS), item 3 -5* (Diener et al. JPA, 1985; 2, 896–906). Depending on the results from the *DSWLS*, the participants were divided into two groups: High or low life quality and group differences were calculated for repetitive symptoms with Student’s t-test. R-Studio (version 2022.07.2) was used for statistical analysis.

**Results:**

In total 86 individuals were included, one individual was excluded due to missing data. Lower life-quality was more frequently reported by females (71%), as compared to males (52%). Mean age was slightly lower in the group with lower life quality (lower: 38.5 years vs. higher: 40.6 years). The group with lower quality of life scored higher on the RBQ-2A questionnaire (mean = 18.6, Standard deviation [SD] = 8.25, n = 54) as compared to the group who reported higher life quality (mean = 13.8, SD = 6.23, n = 32). Higher scores of repetitive behaviors were significantly associated with lower life-quality (*t-value* = 3.0153, degrees of freedom [df] = 78.966, *p-value* = 0.0035).

**Conclusions:**

Lower self-reported life-quality was associated with higher repetitive symptoms in adults diagnosed with ASD. This highlights repetitive symptoms as an important treatment-target when developing pharmacological as well as psychotherapy-oriented treatments for this group of patients.

**Disclosure of Interest:**

None Declared

